# Morphometry and morphology of rostral cranial fossa in brachycephalic dogs – CT studies

**DOI:** 10.1371/journal.pone.0240091

**Published:** 2020-10-01

**Authors:** Wojciech Sokołowski, Karolina Barszcz, Marta Kupczyńska, Michał Czopowicz, Norbert Czubaj, Wojciech Kinda, Zdzisław Kiełbowicz

**Affiliations:** 1 Department of Morphological Sciences, Institute of Veterinary Medicine, Warsaw University of Life Sciences–SGGW, Warsaw, Poland; 2 Division of Veterinary Epidemiology and Economics, Institute of Veterinary Medicine, Warsaw University of Life Sciences–SGGW, Warsaw, Poland; 3 Department of Surgery, Faculty of Veterinary Medicine, Wroclaw University of Environmental and Life Sciences, Wroclaw, Poland; University of Bari, ITALY

## Abstract

Hydrocephalus occurs more often in brachycephalic individuals of different species. Detailed analysis of rostral cranial fossa–region of cerebrospinal fluid outflow–is necessary to understand causes leading to hydrocephalus in specimens with shortened skull. The objective of the study was to determine morphology and morphometry of rostral cranial fossa in brachycephalic dogs. Skulls of 126 dogs of different breeds and morphotypes were examined using computed tomography. Linear and volumetric measurement in the region of rostral cranial fossa and skull base were made. In brachycephalic dogs there is shortening of rostral cranial fossa which is linked with the volume reduction of this region. There are differences in skull base shape between brachycephalic dogs and other morphotypes. Similarities between brachycephalic dogs and patients with craniosynostoses were noted.

## Introduction

Brachycephaly is the shortening of the skull in the anteroposterior dimension [1, 2]. It is a characteristic feature in some breeds of dogs and cats resulting from directed breeding for brachycephaly [3]. In humans, the shortening of the skull in the anterioposterior dimension greater than the norm for a given race is caused either by prolonged recumbency in the supine position and resultant growth disturbances in the infancy period or by pathological premature cranial suture fusion in the so-called “coronal ring” in the course of craniosynostosis [1, 4]. Brachycephalic individuals often suffer from idiopathic hydrocephalus [5–7]. This tendency is observed in some breeds of dogs [5], cats [6] and humans suffering from craniosynostoses (such as the Crouzon or Apert syndrome) [7]. The coexistence of a shortened skull and hydrocephalus in various animal species suggests a mutual relationship, the identification of which may allow a deeper understanding of the causes of excessive accumulation of cerebrospinal fluid (CSF) within the cranial cavity. In our previous study [8], we found that the volume ratio of the rostral cranial fossa (RCF) to the cranial cavity in brachycephalic dogs was significantly smaller than in dogs with other skull morphotypes. The present study is a continuation of previous research and focuses on the topography of the skull base with particular emphasis on the RCF, i.e. the presphenoid bone. The CSF flows from the RCF into the lymphatic system of the nasal cavity through the cribriform plate [9, 10]. A detailed analysis of the anatomical relationships within the RCF may allow to explain the cause of communicating hydrocephalus in some individuals with brachycephaly.

Given that nomenclature used in human and animal anatomy differ, the term “rostral cranial fossa” was used in this study, which is the equivalent of the human “anterior cranial fossa”. The term “brachycephaly” used in relation to dogs and cats signifies morphological variability of the skull consisting of shortening of the anterioposterior dimension of the skull and a resultant increase in the skull index. While it is considered normal for many dog breeds, it is always a pathological condition in humans. Even though, the brachycephalic morphotype in dogs and cats is considered a variant of morphological differentiation, it is associated with various pathologies, such as the brachycephalic obstructive airway syndrome [11].

## Materials and methods

The study was carried out on 126 skulls of dogs of various breeds from 1.5–18 years old. The exact age of 20 animals was unknown, as they were foster pets. However, all the studied animals had been under the care of their owners for a minimum of two years, which confirmed that they were all adult animals with a completed growth phase. The studied samples were obtained from animals that had been euthanised due to illness by veterinary surgeons from the Clinic of Small Animal Diseases of the Warsaw University of Life Sciences and other privately owned veterinary clinics. The owners consented to the euthanasia and the use of animal tissues for the purpose of this study. According to the law in force in Poland, tests on tissues obtained post-mortem do not require an approval of the Ethics Committee (Parliament of the Republic of Poland, 1997). None of the dogs included in this study presented with neurological disorders and none was treated for neurological disease at the time of euthanasia. Following a standard maceration of the heads, computed tomography scans using a Siemens SOMATOM Emotion 16 CT Scanner were performed. The images were obtained using the following protocol: 220 mAs; 130 kV; 1.5 s rotation time; pitch 1.0; and a 0.75 mm final section. Osirix software in bone window view was used to analyse the scans (window width 2000 HU, window level 350 HU).

The distance between the akrokranion–prosthion (AP) was measured in the median plane, and it was assumed that the prosthion was the most rostrally located point within the interincisive suture, while the akrokranion was the most caudally extended point on the external occipital protuberance. The distance between the most laterally extended points on the zygomatic arch (ZyZy) was measured in the frontal plane. The obtained measurements were used to calculate the skull index (SI) = ZyZy/AP x 100 [2]. Based on that index, individuals were classified into one of four groups: dolichocephalic (D) group–SI ≤ 50.00, mesaticephalic 1 (M1) group–SI = 50.01 to 65.00, mesaticephalic 2 (M2) group–SI– 65.01 to 80.00 and brachycephalic (B) group–SI > 80.00. The dogs classified as mesaticephalic were divided into two subgroups–M1 and M2, as it was assumed that there might be morphological differences between individuals with lower and higher skull indices. Table 1 presents the number of dogs of each breed included in the study.

**Table 1:** Number of dogs of each breed.

No.	Breed	Number of dogs
1	American Staffordshire Terrier	9
2	Australian Shepherd	2
3	Bavarian Mountain Hound	1
4	Beagle	2
5	Bernard	1
6	Bernese Mountain Dog	2
7	Black Russian terrier	3
8	Boxer	8
9	Bull Terrier	2
10	Cavalier King Charles Spaniel	1
11	Central Asian Shepherd	1
12	Dachshund	7
13	Dalmatian	2
14	Doberman Pinscher	2
15	English Bulldog	4
16	English Cocker Spaniel	1
17	English Springer Spaniel	1
18	French Bulldog	9
19	German Shepherd	17
20	German Wirehaired Pointer	1
21	Golden Retriever	1
22	Gordon Setter	1
23	Great Dane	2
24	Italian Mastiff	4
25	Labrador Retriever	6
26	Miniature Pinscher	4
27	Miniature Poodle	1
28	Newfoundland	1
29	Pekingese	6
30	Rottweiler	5
31	Russian Wolfhound	1
32	Samoyed	1
33	Schipperke	1
34	Scottish Terrier	1
35	Shih Tzu	1
36	Siberian Husky	2
37	Standard Schnauzer	1
38	Welsh Terrier	1
39	White Shepherd	1
40	Wire-Haired Fox Terrier	1
41	Yorkshire Terrier	8

**Fig 1:**
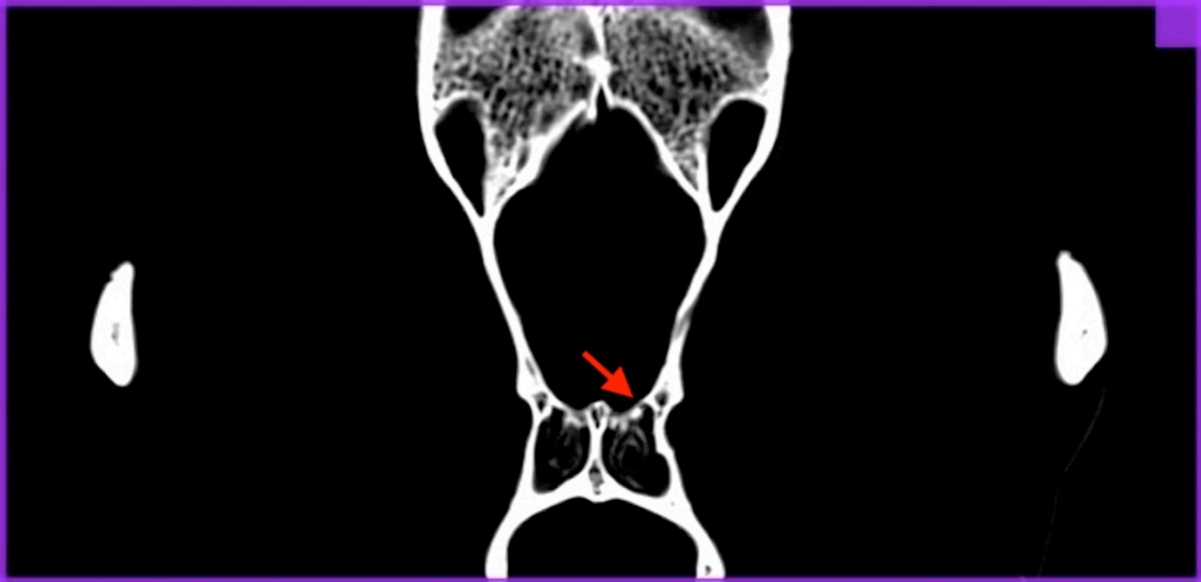
Determining of the rostral border of the presphenoid bone point (RPB). Position of the most caudal cribriform foramina on the transverse scan. Cribriform foramen is marked with arrow.

**Fig 2:**
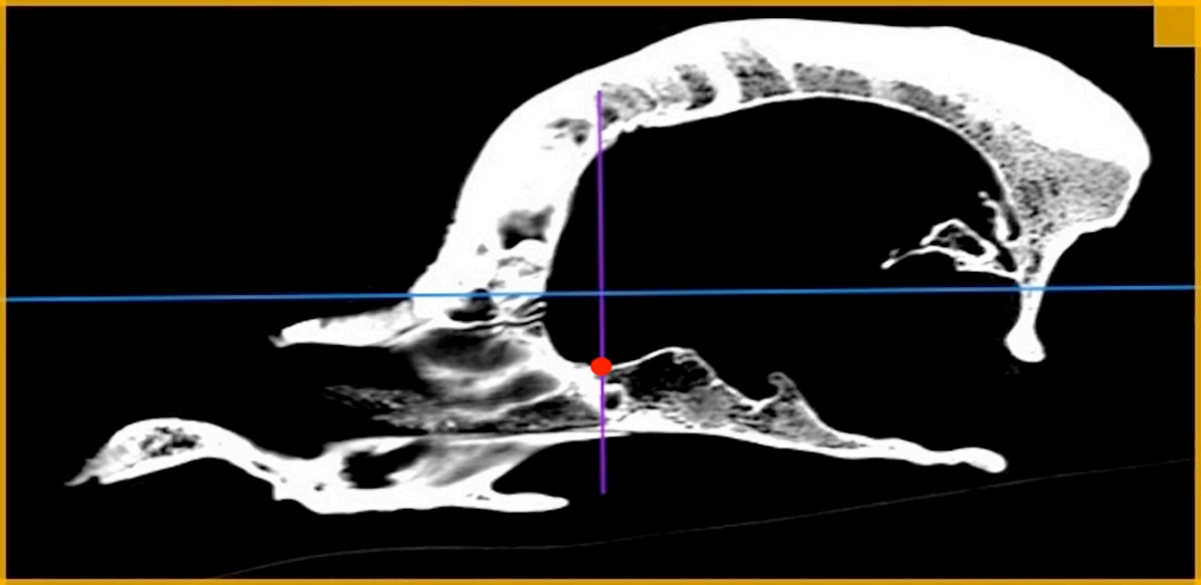
Determining of the rostral border of the presphenoid bone point (RPB). Position of RPB point on sagittal scan is marked with a dot.

Subsequent measurement points were determined in the median plane using scans in the transverse plane. The rostral border of the presphenoid bone point (RPB) was located at the level of the caudal border of the cribriform foramina of the cribriform plate. The position of the most caudally located cribriform foramen was determined in the transverse plane (Fig 1). The location of the RPB was then determined on a scan in the median plane on the inner surface of the base of the skull at the level of intersection with the line corresponding to the mentioned transverse plane (Fig 2). The sulcus chiasmatis point (SC) was the most caudally projected point of the chiasmatic groove. The caudal border of the chiasmatic groove was determined on the scans in the transverse plane. The same principles as for determining the RPB were used to establish the SC. The rostral border of the basis of the presphenoid bone wings (RBPW) determined the position of the caudal border of the ethmoidal foramina. The location of this structure was determined on the scans in the transverse plane. In the case of asymmetric foramina, the foramina located more caudally were used. RBPW was determined on inner surface of the base of the skull using the same principles as in the case of RPB. The hypophyseal fossa point (HF) was the most ventral point of the hypophyseal fossa. In some individuals, it was difficult to assess the HF due to the morphological variability of the hypophyseal fossa. Hence, in all the animals, HF was always determined at the level of the rostral border of the caudal alar foramina, which were defined using scans in the transverse plane. HF was established by analogy to the RPB.

**Fig 3:**
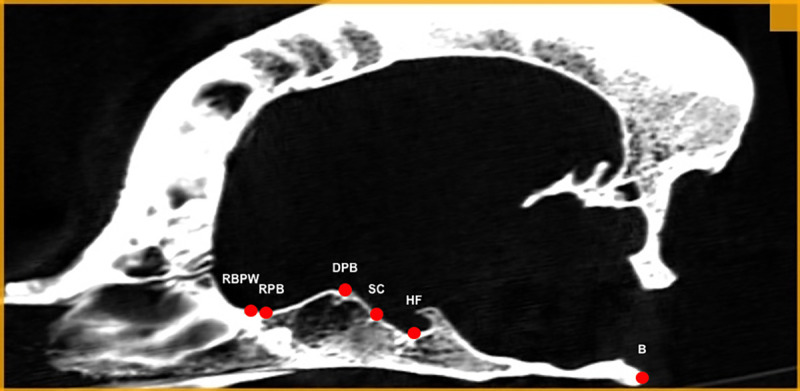
Points determinated in the median plane. RPB–rostral border of the presphenoid bone point, SC–sulchus chiasmatis point, RBPW–rostral border of the basis of the presphenoid bone wings, HF–hypophyseal fossa point, DPB–dorsal aspect of the presphenoid bone point and B–basion.

The following points were determined in the median plane. The dorsal aspect of the presphenoid bone point (DPB) was the most dorsally extended point of the body of the presphenoid bone. The basion (B) was the most caudally extended point of the basilar part of the occipital bone. The location of all the points on the scan in the median plane is presented in Fig 3.

Linear measurements were also taken in the median plane. The presphenoid bone length (PBL) was measured between RPB and SC. The length of the basis of the presphenoid bone wings (PBWL) was measured between RBPW and SC. The skull base length (SBL) was measured between RPB and B. The values of PBL, PBWL and SBL were calculated for all the animals.

Two volumetric measurements, corresponding to the dimensions of the cranial cavity (volCC) and the volume of the rostral cranial fossa (volRCF) were collected using previously published protocol [8]. Due to insufficient development of the bony protrusion being caudal border of the RCF the volRCF and volCC measurements and statistical analysis were performed on 117 animals (three Boxer dogs, a Bull Terrier, Dachshund, Labrador Retriever, Miniature Pinscher, Walsh Terrier and Yorkshire Terrier were excluded).

**Fig 4:**
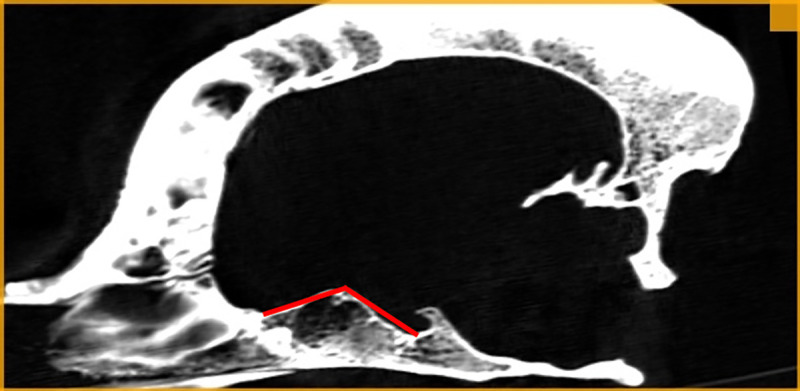
RMCFA–rostral / medial cranial fossa angle.

The values of the rostral / middle cranial fossa angle (RMCFA) were analysed between two rays in the median plane. The first ray was the line between RPB and DPB. The second ray was the line between DPB and HF. The tip of the resultant angle was the most dorsally located point of the RCF (Fig 4). A Central Asian Shepherd and French Bulldog were excluded from the analysis due to an unusual shape of the pituitary fossa. Hence, RMCFA were calculated in and statistical analyses were performed on 124 animals.

An objective comparison and analysis of the collected linear measurements (absolute data) are not possible due to the morphological variations of the skulls between the studied dogs. In craniometry this is usually eliminated using indices. They determine the mutual relations between the absolute data. In the performed analyses, the following indices were developed: I 1 = PBL/SBL, I 2 = PBWL/SBL, I 3 = PBL/PBWL and I 4 = volRCF/volCC.

Linear and volumetric measurements of each individual are listed in S1 Table.

### Statistical methods

Categorical variables were expressed in counts and percentages and were compared between the groups using the Pearson’s chi-square test. Numerical variables were presented as the arithmetic mean, standard deviation (SD), the range or the arithmetic mean and 95% confidence interval (CI 95%) in figures. Numerical variables were compared between two groups using the unpaired Students t-test. If more than two groups were compared, a one-way ANOVA with Tukey HSD post-hoc test for unequal sample sizes or Welch ANOVA with Games-Howell post-hoc test were used, depending on the result of the Brown-Forsythe test for homogeneity of variances. A linear correlation between numerical variables was assessed using scatter plots and the Pearson’s product-moment correlation coefficient (r) with CI 95%. All the tests were two-sided. A significance level (α) was set at 0.05. Statistical analysis was performed in Statistica 12.5 (StatSoft Inc., Tulsa, OK) and IBM SPSS Statistics 24. Plots were prepared in TIBCO Statistica 13.3.0 (TIBCO Software Inc., Palo Alto, CA).

## Results

**Fig 5:**
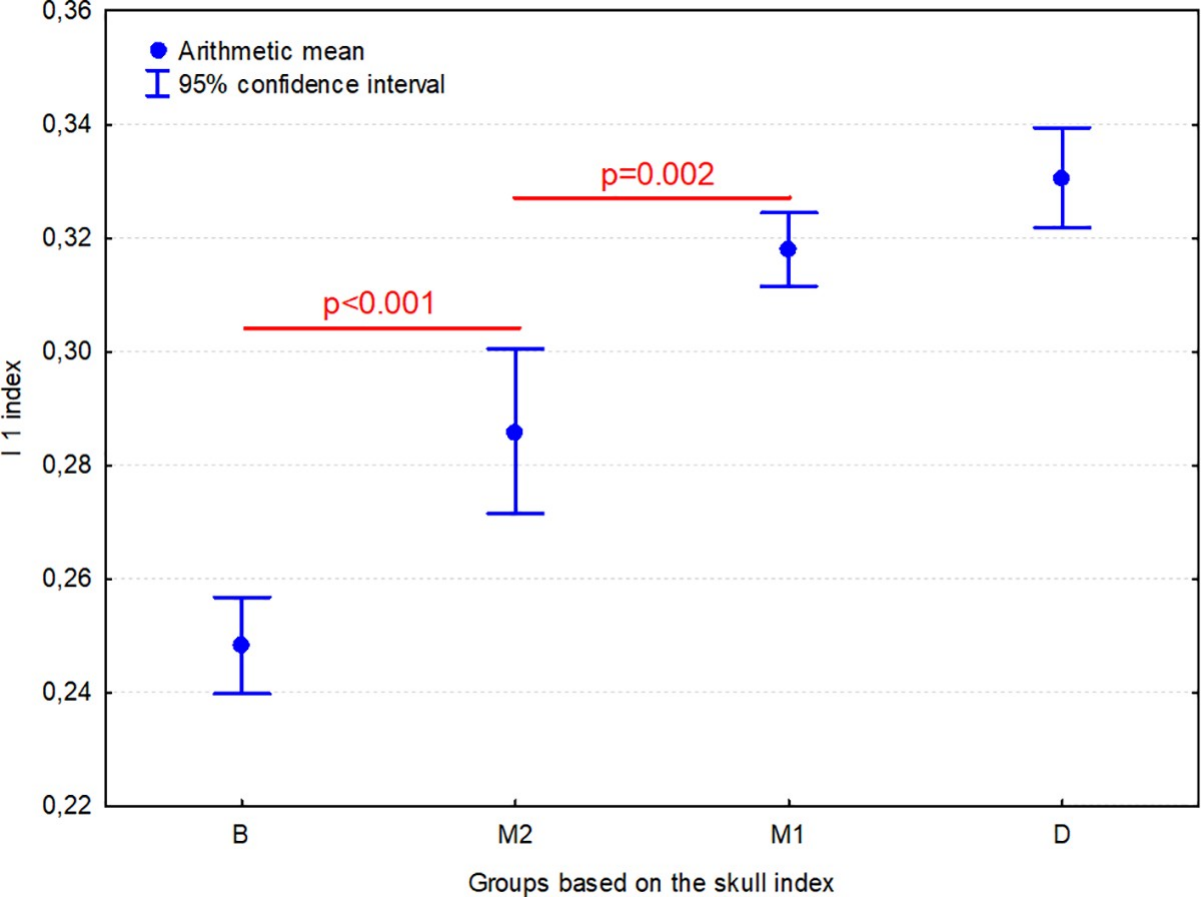
Comparison of I 1 index (PBL/SBL) between 4 groups of dogs. Individuals are classified according to their skull index (SI): B–brachycephalic group (SI >80.00), M1 –mesaticephalic 1 group (SI from 50.01 to 65.00), M2 –mesaticephalic 2 group (SI from 65.01 to 80.00); D–dolichocephalic group (SI ≤50.00). Horizontal lines indicate groups which differ significantly from each other.

The I 1 index in group B was significantly lower (p<0.001) than in group M1, M2 and D. In addition, this index in group M2 was significantly lower than in group M1 (p = 0.002) (Fig 5). This means that as the SI increases, the presphenoid bone contributes less to the skull base, reaching the lowest values in brachycephalic dogs.

The statistical analysis of I 2 index did not reveal significant differences between groups. This indicates that changes in the SI are not associated with changes in the length of the base of the presphenoid wings.

**Fig 6:**
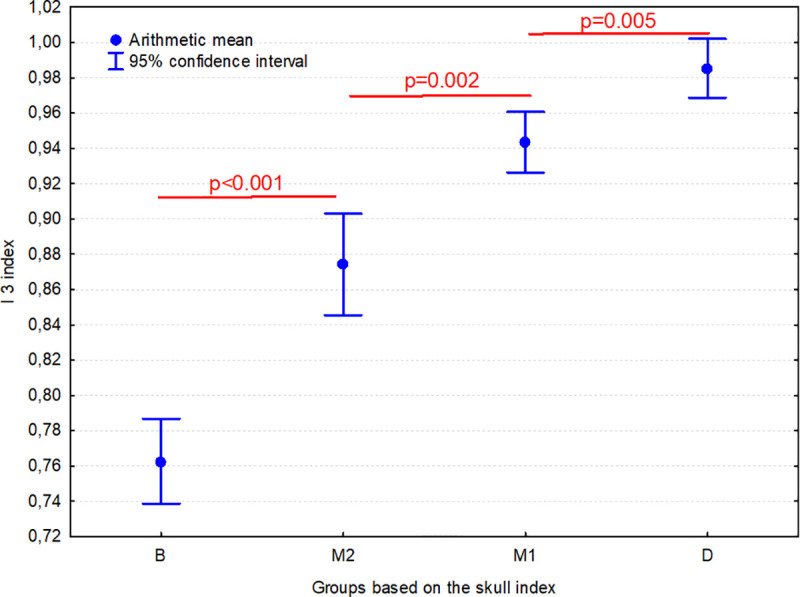
Comparison of I 3 index (PBL/PBWL) between 4 groups of dogs. Individuals are classified according to their skull index (SI): B–brachycephalic group (SI >80.00), M1 –mesaticephalic 1 group (SI from 50.01 to 65.00), M2 –mesaticephalic 2 group (SI from 65.01 to 80.00); D–dolichocephalic group (SI ≤50.00). Horizontal lines indicate groups which differ significantly from each other.

The I 3 index in group B was significantly lower (p<0.001) than in group M1, M2 and D. Moreover, this index was also significantly lower in the M2 group than in the M1 group (p = 0.002), while it was significantly lower in the M1 group than in group D (p = 0.005) (Fig 6). This indicates that as the SI increases, there is a shortening of the body of the presphenoid bone relative to the base of the presphenoid bone wings.

**Fig 7:**
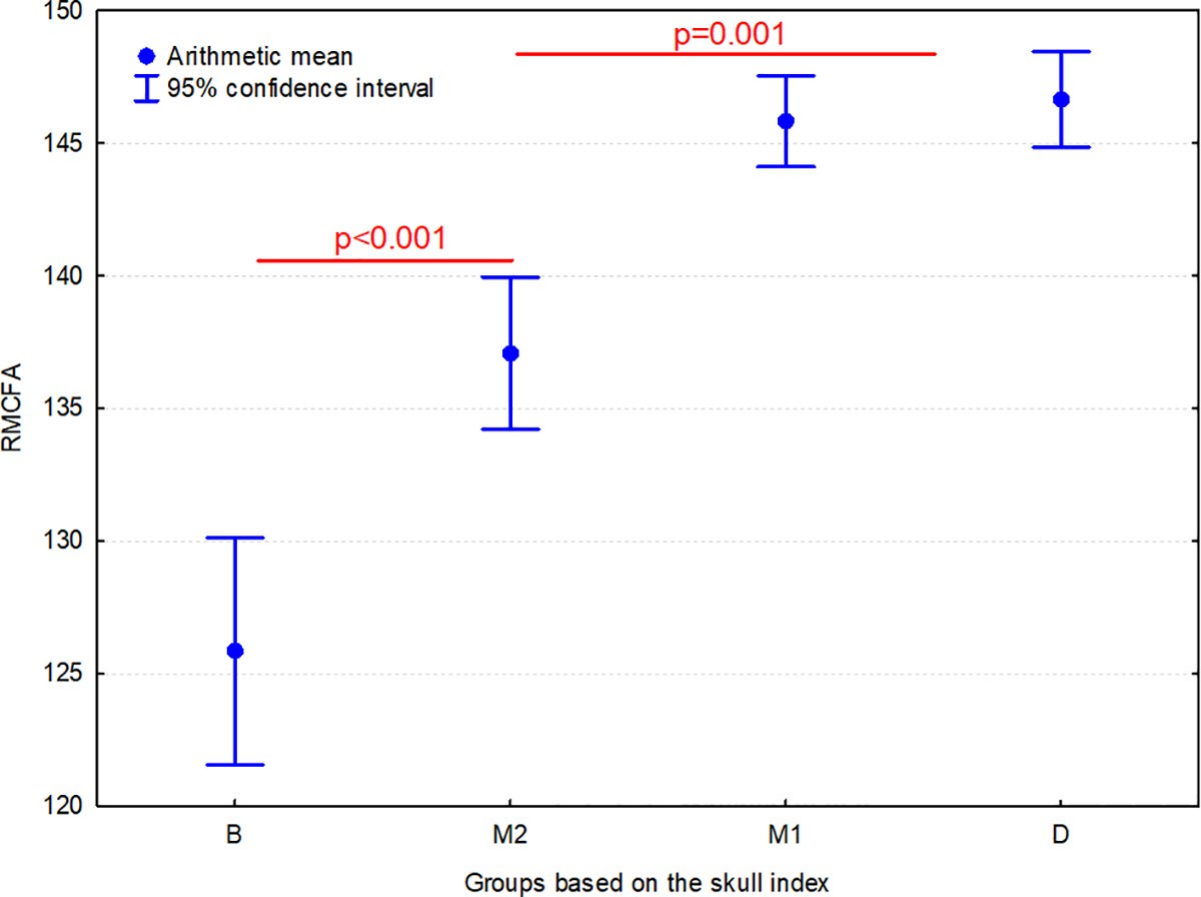
Comparison of the rostral / middle cranial fossa angle (RMCFA) between 4 groups of dogs. Individuals are classified according to their skull index (SI): B–brachycephalic group (SI >80.00), M1 –mesaticephalic 1 group (SI from 50.01 to 65.00), M2 –mesaticephalic 2 group (SI from 65.01 to 80.00); D–dolichocephalic group (SI ≤50.00). Horizontal lines indicate groups which differ significantly from each other.

The value of RMCFA in group B was significantly lower (p<0.001) than in group M1, M2 and D. In addition, the value of RMCFA in group M2 group was significantly lower than in group M1 (p = 0.001) and group D (p<0.001) (Fig 7). Hence, as the SI increases, there is a dorsal shift of the RCF relative to the middle cranial fossa and/or the ethmoidal fossa.

**Fig 8:**
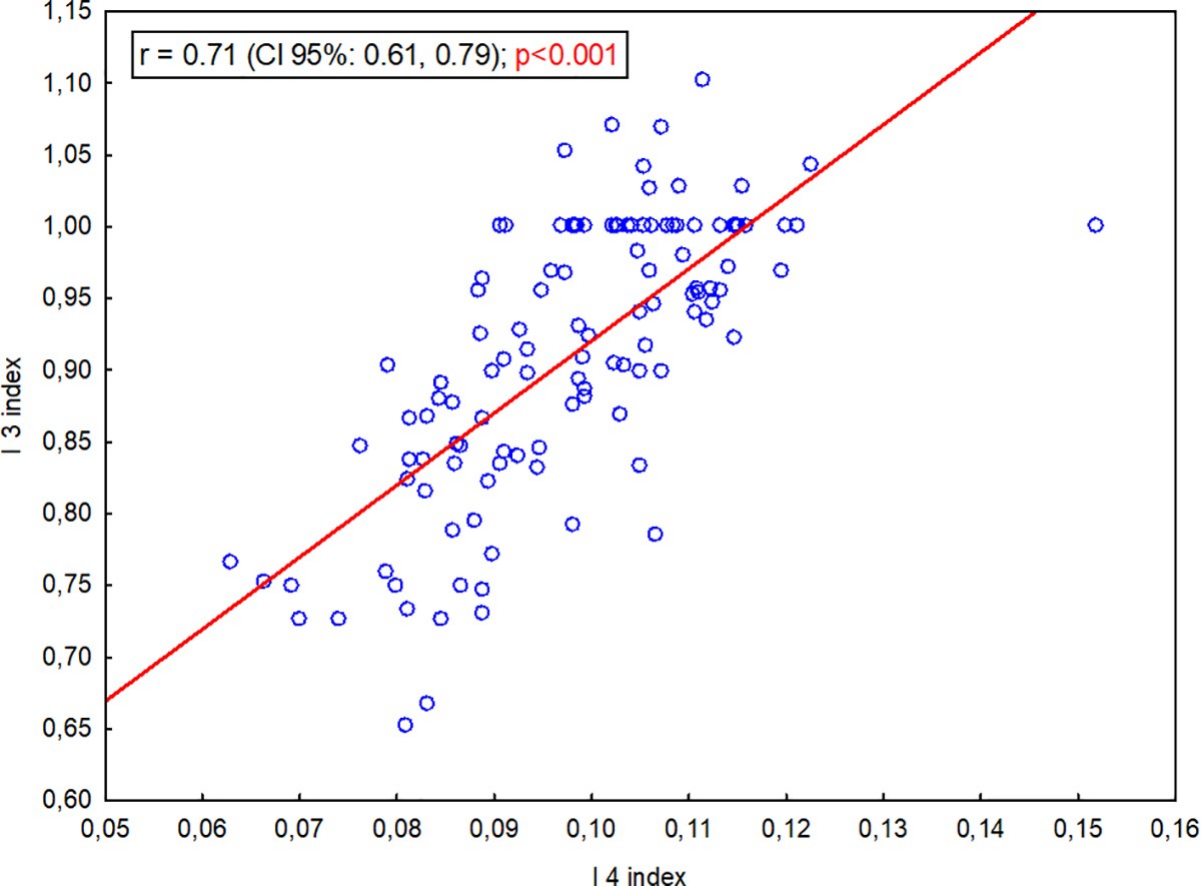
Correlation between the I 3 index (PBL/PBWL) and the I 4 index (volRCF/volCC).

**Fig 9:**
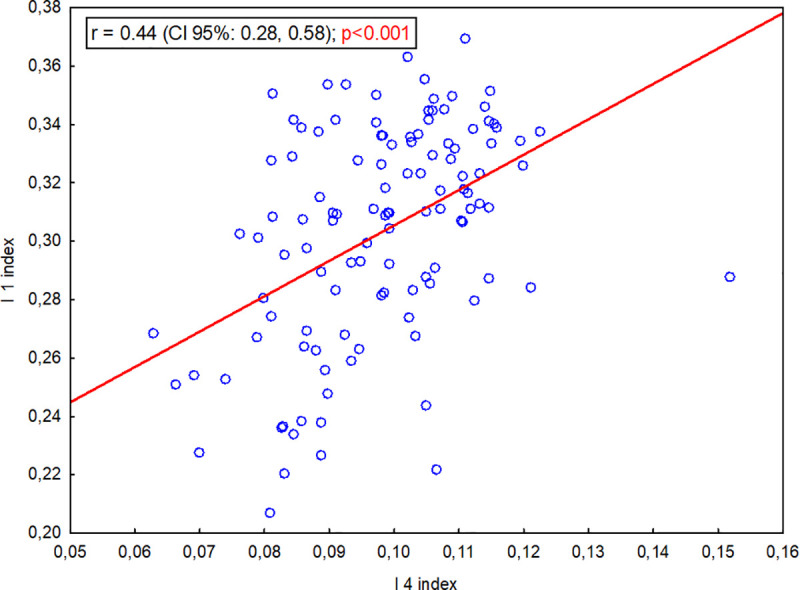
Correlation between the I 1 index (PBL/SBL) and the I 4 index (volRCF/volCC).

As the I 4 volumetric index increased, the I 3 index also increased (significant strong positive correlation, r = 0.71, p<0.001; Fig 8). As the I 4 length index increased, the I 1 index increased (significant weak positive correlation;, r = 0.44, p<0.001; Fig 9). This shows that as the body of the presphenoid bone shortened in relation to the basis of the presphenoid bone wings and the skull base, the volume of the RCF decreased in relation to the entire cranial cavity.

## Discussion

Hydrocephalus is observed frequently in brachycephalic dog breeds [5]. The Boston Terrier, English Bulldog, Lhasa Apso, Pekingese and Pug are considered predisposed to congenital hydrocephalus [5]. Studies of Persian cats, a typically brachycephalic breed, indicate more frequent congenital hydrocephalus in individuals with shorter facial skeleton [6]. Hydrocephalus is a common finding in children suffering from craniosynostoses, which leads to the development of brachycephaly as a result of premature closure or fusion of some sutures and synchondroses respectively [7]. Although the causes of hydrocephalus and disturbed CSF circulation have been widely studied, their pathomechanism still remains unclear [12]. The co-occurrence of shortening of the skull and hydrocephalus in various species is interesting and warrants further investigation of possible causes of this association. One experimental study in rats suggests that impaired CSF circulation in the skull base region and its outflow through the cribriform plate are associated with hydrocephalus [10]. For that reason it seems important to analyse the anatomical relations of the RCF and the structure of the skull base in brachycephalic individuals.

### Rostral cranial fossa

The cribriform foramina, which limit the RCF, are considered the site of CSF drainage into nasal lymphatic vessels [2, 9]. The CSF drains from the subarachnoid space into the nasal cavity via the perineural space of the olfactory nerve [13]. Studies performed on rats [10] and sheep [14] suggest that blockage of this pathway of CSF flow may lead to hydrocephalus or increased intracranial pressure. The authors’ study performed on 71 animals strongly suggests that the ratio of the volume of the RCF to the skull is lower in brachycephalic dogs than in dogs with other skull types. Based on those findings, the authors postulated that a decrease of the RCF volume may cause overcrowding of the brain structures it contains [8]. However, this hypothesis needs to be verified. It is supported by observations in Persian cats, whereby animals with a shortened facial skeleton suffered from compression on the rostral aspect of the brain with a simultaneous change in the brain shape and ventral shift of the olfactory bulbs [6]. The results presented in this study clearly indicate significant differences in the shape of the presphenoid bone that forms the base of the RCF in dogs with various morphotypes. The aforementioned data suggest that as the values of the SI increase, there is a gradual, statistically significant shortening of the body of the presphenoid bone, both in relation to the base of the presphenoid wings and to the entire skull base. Furthermore, a decrease in the volume of the RCF relative to the entire cranial cavity concurrent with the shortening of the body of the presphenoid bone were observed. These data imply that disturbances in the rostro-caudal growth of the body of the presphenoid bone may occur in brachycephalic dogs, leading to a reduced volume of the nasal cavity. Interestingly, shortening of the RCF is also a feature of the Crouzon and the Apert syndromes in humans [15]. Elongation of the presphenoid bone results from the activity of two synchondroses–the sphenoethmoidal synchondrosis and the intersphenoid synchondrosis [16]. In the course of Crouzon and Apert syndromes, there is a premature closure of the sutures and synchondroses of so-called “coronal ring” [15, 17–19], which include the frontoparietal suture, frontosphenoid suture and the sphenoethmoidal synchondrosis [18]. Premature ossification of these structures leads to brachycephaly and shortening of the RCF [20]. In people, the intersphenoid synchondrosis physiologically closes in the prenatal period [21]. In contrast, the growth of the presphenoid bone in dogs is still poorly understood, and the time of closure of the sphenoethmoidal synchondrosis remains unknown. Two studies have so far been published on this topic [16, 22]. The first is based on MRI studies of 174 dogs and suggests that the intersphenoid synchondrosis remains open until the age of 18 months [16]. The second study analysed 134 skulls of adult dogs of various breeds and found that there was a higher percentage of closed intersphenoid synchondroses in bulldog-like breeds than in other breeds of dogs [22]. Due to scarce information on this topic, it is difficult to draw conclusions on the time of the closure of this synchondrosis in dogs with various skull morphotypes. It is interesting that the body of the presphenoid bone may be shortened in dogs despite the late closure of the intersphenoid synchondrosis. Thereby it can be hypothesized that as in humans, premature closure of the sphenoethmoidal synchondrosis may have a crucial impact on the growth of this structure. Alternatively, the shortening of the body of the presphenoid bone in dogs may result from a disturbed function of persistently open synchondroses. Regardless of its pathomechanisms shortening of the RCF seems to be common in brachycephalic breeds of dogs and in people with the Crouzon and Apert syndrome. In the authors’ opinion, the concept of potential crowding of the brain structures within the RCF and possible CSF outflow alterations requires further analysis in brachycephalic dogs and people with craniosynostoses.

Dogs of various age were enrolled in our study. In young individuals, the synchondroses, such as that of the presphenoid bone, may have not undergone full closure yet. To fully understand the differences in the development of the presphenoid bone, the base of the RCF and the entire skull base, further studies on growing individuals of various breeds and morphotypes in similar age groups are warranted. The animals included in this study had no neurological deficits and other symptoms characteristic of hydrocephalus, such as a dome-shaped skull. Hence, there may be differences in time of synchondroses closure and the formation of the skull base between clinically healthy brachycephalic dogs and those with congenital idiopathic hydrocephalus.

### Skull base deformities

Based on the traditional concept of CSF circulation, CSF must flow a certain distance from the site of production before it reaches the cribiform plate and the perineural space of the olfactory nerve [23, 24]. The observations on rats indicate that a considerable part of the CSF flows rostrally along the base of the skull to the cribriform plate [23, 25, 26] and impairment of this flow at the level of the basal cisterns leads to hydrocephalus [10]. The severity of ventricular enlargement is proportional to the degree of impaired CSF flow through cribiform plate [10]. Similarly, surgeries at the base of the skull in humans that lead to scarring in this region result in hydrocephalus in roughly 1 out of ten patients [27]. It may be assumed that specific deformities of the skull base region may, hypothetically, cause disturbances in the CSF flow. The RMCFA angle in dogs with a shortened skull is much lower than in other dogs. That means that the DPB point, which corresponds to the RCF, is shifted dorsally with respect to the cribiform plate and the middle cranial fossa. Interestingly, similar observations were made in humans with Apert syndrome, where an elevation of the sphenoid ridge, which forms the border between the cranial and middle cranial fossa, was observed [28].

### Conclusions

Our results indicate that there is shortening and dorsal shifting of the body of presphenoid bone in brachycephalic dog individuals. Shortening of presphenoid bone is correlated with RCF volume reduction. Some of these observations are similar to those made in humans with Crouzon and Apert syndromes. Mentioned parallels and more frequent occurrence of hydrocephalus in individuals with shortened skull, encourages further research on CSF circulation in RCF region.

## Supporting information

S1 Table Linear and volumetric measurements of the individuals. (XLS)

## References

[1] Graham JM Jr, Sanchez-Lara PA. Smith's recognizable patterns of human deformation. Elsevier Health Sciences; 2015.

[2] Evans HE, De Lahunta A. Miller's anatomy of the dog-E-Book. Elsevier Health Sciences; 2013.

[3] Schoenebeck JJ, Ostrander EA. The genetics of canine skull shape variation. Genetics. 2013;193(2):317–25. 10.1534/genetics.112.145284 23396475 PMC3567726

[4] Allanson JE, Cunniff C, Hoyme HE, McGaughran J, Muenke M, Neri G. Elements of morphology: standard terminology for the head and face. Am J Med Genet A. 2009;149(1):6–28. 10.1002/ajmg.a.32612 PMC2778021 19125436

[5] Selby LA, Hayes HM Jr, Becker SV. Epizootiologic features of canine hydrocephalus. Am J Vet Res. 1979;40(3):411–413. 475097

[6] Schmidt MJ, Kampschulte M, Enderlein S, Gorgas D, Lang J, Ludewig E, et al The relationship between brachycephalic head features in modern persian cats and dysmorphologies of the skull and internal hydrocephalus. J Vet Intern Med. 2017;31(5):1487–501. 10.1111/jvim.14805 28833532 PMC5598898

[7] Cinalli G, Sainte-Rose C, Kollar EM, Zerah M, Brunelle F, Chumas P, et al Hydrocephalus and craniosynostosis. J neurosurg. 1998;88(2):209–14. 10.3171/jns.1998.88.2.0209 9452225

[8] Sokołowski W, Czubaj N, Skibniewski M, Barszcz K, Kupczyńska M, Kinda W, et al Rostral cranial fossa as a site for cerebrospinal fluid drainage–volumetric studies in dog breeds of different size and morphotype. BMC Vet Res. 2018;14(1):162 10.1186/s12917-018-1483-3 29776403 PMC5960198

[9] Johnston M, Zakharov A, Papaiconomou C, Salmasi G, Armstrong D. Evidence of connections between cerebrospinal fluid and nasal lymphatic vessels in humans, non-human primates and other mammalian species. Cerebrospinal fluid res. 2004;1(1):2 10.1186/1743-8454-1-2 15679948 PMC546409

[10] Nagra G, Li J, McAllister JP, Miller J, Wagshul M, Johnston M. Impaired lymphatic cerebrospinal fluid absorption in a rat model of kaolin-induced communicating hydrocephalus. Am J Physiol Regul Integr Comp Physiol. 2008;294(5)1752-9. 10.1152/ajpregu.00748.2007 18305019

[11] Packer RM, Hendricks A, Tivers MS, Burn CC. Impact of facial conformation on canine health: brachycephalic obstructive airway syndrome. PLoS One. 2015;10(10):e0137496 10.1371/journal.pone.0137496 26509577 PMC4624979

[12] Collmann H, Sörensen N, Krauss J. Hydrocephalus in craniosynostosis: a review. Childs Nerv Syst. 2005;21(10):902–12. 10.1007/s00381-004-1116-y 15864600

[13] Erlich SS, McComb JG, Hyman S, Weiss MH. Ultrastructural morphology of the olfactory pathway for cerebrospinal fluid drainage in the rabbit. J neurosurg. 1986;64(3):466–73. 10.3171/jns.1986.64.3.0466 3950724

[14] Mollanji R, Bozanovic-Sosic R, Silver I, Li B, Kim C, Midha R, et al Intracranial pressure accommodation is impaired by blocking pathways leading to extracranial lymphatics. Am J Physiol Regul Integr Comp Physiol. 2001;280(5):1573–81. 10.1152/ajpregu.2001.280.5.R1573 11294783

[15] Cohen MM Jr, Kreiborg S. A clinical study of the craniofacial features in Apert syndrome. Int J Oral Maxillofac Surg. 1996;25(1):45–53. 10.1016/s0901-5027(96)80011-7 8833300

[16] Schmidt MJ, Volk H, Klingler M, Failing K, Kramer M, Ondreka N. Comparison of closure times for cranial base synchondroses in mesaticephalic, brachycephalic, and cavalier king charles spaniel dogs. Vet Radiol Ultrasound. 2013;54(5):497–503. 23782353 10.1111/vru.12072

[17] Das DK, Hota S, Mahapatro S. Apert Syndrome with Epibulbar Dermoid Cyst in the Eye-Coincidence or an Additional Clinical Finding: A Case Report. Indian Journal of Clinical Practice. 2014;25(7):680–2.

[18] Hupp JR, Tucker MR, Ellis E. Oral and Maxillofacial Surgery-E-Book. Elsevier Health Sciences; 2017.

[19] Burdi AR, Kusnetz AB, Venes JL, Gebarski SS. The natural history and pathogenesis of the cranial coronal ring articulations: implications in understanding the pathogenesis of the Crouzon craniostenotic defects. Cleft Palate J. 1986;23(1):28–39. 3455900

[20] Blaser SI, Padfield N, Chitayat D, Forrest CR. Skull base development and craniosynostosis. Pediatr Radiol. 2015;45 Suppl 3:S485–96. 26346154 10.1007/s00247-015-3320-1

[21] Hayashi I. Morphological relationship between the cranial base and dentofacial complex obtained by reconstructive computer tomographic images. Eur J Orthod. 2003;25(4):385–91. 10.1093/ejo/25.4.385 12938845

[22] Geiger M, Haussman S. Cranial suture closure in domestic dog breeds and its relationships to skull morphology. Anat Rec. 2016;299(4):412–20. 10.1002/ar.23313 26995336

[23] Brinker T, Stopa E, Morrison J, Klinge P. A new look at cerebrospinal fluid circulation. Fluids Barriers CNS. 2014;11(1):10 24817998 10.1186/2045-8118-11-10 PMC4016637

[24] Sokołowski W, Barszcz K, Kupczyńska M, Czubaj N, Skibniewski M, Purzyc H. Lymphatic drainage of cerebrospinal fluid in mammals–are arachnoid granulations the main route of cerebrospinal fluid outflow?. Biologia. 2018;73(6):563–8. 10.2478/s11756-018-0074-x 30147112 PMC6097054

[25] Ghersi-Egea JF, Finnegan W, Chen JL, Fenstermacher JD. Rapid distribution of intraventricularly administered sucrose into cerebrospinal fluid cisterns via subarachnoid velae in rat. Neuroscience. 1996;75(4):1271–88. 10.1016/0306-4522(96)00281-3 8938759

[26] Kida S, Pantazis AWRO, Weller RO. CSF drains directly from the subarachnoid space into nasal lymphatics in the rat. Neuropathol Appl Neurobiol. 1993;19(6):480–8. 10.1111/j.1365-2990.1993.tb00476.x 7510047

[27] Duong DH, O'Malley S, Sekhar LN, Wright DG. Postoperative hydrocephalus in cranial base surgery. Skull Base Surg. 2000;10(4):197–200. 10.1055/s-2000-9331 17171147 PMC1656863

[28] Tokumaru AM, Barkovich AJ, Ciricillo SF, Edwards MS. Skull base and calvarial deformities: association with intracranial changes in craniofacial syndromes. AJNR Am J Neuroradiol. 1996;17(4):619–30. American Journal of Neuroradiology 17.4 (1996): 619–30. 8730180 PMC8337260

